# Examining innovation in hospital units: a complex adaptive systems approach

**DOI:** 10.1186/s12913-020-05403-2

**Published:** 2020-06-18

**Authors:** Wiljeana Jackson Glover, Noa Nissinboim, Eitan Naveh

**Affiliations:** 1 Technology, Operations, and Information Management Division, Babson College, Babson Park, Wellesley, MA 02457 USA; 2 Industrial Engineering and Management, Technion – Israel Institute of Technology, Haifa, Israel

**Keywords:** Innovation, Complexity, Healthcare

## Abstract

**Background:**

We are in an innovation age for healthcare delivery. Some note that the complexity of healthcare delivery may make innovation in this setting more difficult and may require more adaptive solutions. The aim of this study is to examine the relationship between unit complexity and innovation, using a complex adaptive systems approach in a hospital setting.

**Methods:**

We conducted a quantitative study of 31 hospital units within one hospital and use complex adaptive systems (CAS) theory to examine how two CAS factors, autonomy and performance orientation, moderate the relationship between unit complexity and innovation.

**Results:**

We find that unit complexity is associated with higher innovation performance when autonomy is low rather than high. We also find that unit complexity is associated with higher innovation performance when performance orientation is high rather than low. Our findings make three distinct contributions: we quantify the influence of complexity on innovation success in the health care sector, we examine the impact of autonomy on innovation in health care, and we are the first to examine performance orientation on innovation in health care.

**Conclusions:**

This study tackles the long debate about the influence of complexity on healthcare delivery, particularly innovation. Instead of being subject to the influence of complexity with no means of making progress or gaining control, hospitals looking to implement innovation programs should provide guidance to teams and departments regarding the type of innovation sought and provide support in terms of time and management commitment. Hospitals should also find ways to promote and make successful pilot implementations of such innovations visible in the organization. A close connection between the targeted innovation and the overall success and performance of the hospital unit is ideal.

## Background

The forces of innovation mold the evolutionary pattern of an industry’s state of technology and advancement [1]. These forces in the health care industry have driven radical scientific advancements and medical treatment innovations for decades. We are also entering an innovation age for hospital care delivery, with significant investments being made to drive hosptital innovation, e.g., [2]. We define hospital unit innovation as the initiation, implementation, and use of new work processes and methods as well as the adaptation of new technologies [3]. Medical and service units within hospitals are generating, implementing and adapting new ideas to their settings, thus innovating their processes, clinical and administrative methods, and technology usage [4, 5].

To explore how hospital unit innovation is achieved, we must understand if the phenomenon is best categorized as simple, complicated, complex, or chaotic [6]. Simple phenomena are easily understood and done; there are rules or best practices in place, few interconnecting parts, and great internal and external stability. Complicated phenomena consist of many interconnecting parts or elements; however there are still a range of correct answers. Complex phenomena consist of many different and interconnected parts, making it difficult, and sometimes impossible to predict how the system would operate based on the understanding of the system’s separate parts. Thus for complex systems, cause and effect relationships can only be deduced in retrospect, and there are no right answers. Finally, chaotic phenomena, is a state of complete confusion and disorder, means that cause and effect relationships are unclear, and events are too confusing to wait for a knowledge-based response.

As Plesk [7] notes, “Innovation in health care is not a complicated issue. It is a complex issue. (p. 2)” The complexity of care has skyrocketed, with more clinical prevention, diagnoses, and treatment options, increased interdisciplinary care, and more interconnected stakeholders [8]. Hospital units are complex such that they cannot be fully understood through linear thinking alone. Characteristics that further support describing hospital units as complex include: (a) team members are interdependent, (b) interactions between team members can produce unpredictable behavior and can generate new behavior, (c) it is impossible to always predict the behavior resulting from the interactions, and (d) small changes in variables can have small impacts at some times, and large impacts under other conditions [9, 10]. In such conditions, cause and effect relationships of team members innovations can in many times only be deduced in retrospect. Because both health care innovation hospital unit teams and have been characterized as complex, we describe hospital unit innovation as a complex phenomenon.

Greater complexity may be significantly and positively associated with innovative behavior, but complexity is rarely considered in theoretical models of healthcare innovation [11]. To address this theoretical gap, we use complex adaptive systems theory to examine innovation within hospital units. A complex adaptive system is a collection of individual agents with freedom to act in ways that are often unpredictable, and whose actions are interconnected so that one agent’s actions changes the context for other agents [8]. Clinical practice, organization, and information management within hospitals are interdependent and built around multiple self-adjusting and interacting systems and thus are best described as complex adaptive systems.

Innovation within complex adaptive systems, e.g., hospital units, requires a different approach than in complicated systems, e.g., building a rocket. Complicated systems can be mechanistically broken down into component parts and detailed plans can be defined for achieving innovation [12]. However, a mechanistic approach that is sufficient for complicated systems yields limited success for innovation in complex adaptive systems (CAS). Instead, the emergent behaviors and agents’ attitudes toward change or innovation may be critical for successful innovations in CAS like hospital units.

Leaders within CAS must determine the right balance of emergent versus controlled behavior. Emergent patterns allow for autonomous action, innovation and flexibility; conversely, allowing too much emergence can undermine managerial predictability and work routines [13]. The general innovation literature suggests that more emergent behavior via more autonomy may be ideal [14]. However, minimum specifications, e.g., boundaries, resources, direction pointing, and permissions, may provide the appropriate guidance for hospital units to develop and adopt innovations within CAS [12]. We specifically examine the role of autonomy on the relationship between complexity and hospital unit innovation. Scholars note that levels of autonomy needed to achieve innovation may vary depending on the levels of complexity within the organization [15]. We examine the role of autonomy to moderate the relationship between complexity and innovation within hospital units.

Innovation within CAS also requires that we account for the attitudes and perceptions of the agents within the adopting unit or system [11, 16]. CAS theory suggests that the attitudes and motivations for influencing teams and departments within complex systems are different that in complicated systems. Instead of financial incentives, teams may be more likely to respond or be “attracted” to innovation by showing they can perform well and prove their abilities to others within the context of their local needs [12]. We examine the role of performance orientation to moderate the relationship between complexity and innovation within hospital units.

In summary, we explore the relationship between unit complexity, autonomy, and performance orientation on innovation within 31 hospital units. Our research has two major contributions. First, we apply CAS to hospital unit innovation. The characteristics of complexity suggest that complexity is a continuous and not dichotomous phenomena [9]. For example, referring to the complexity characteristics described above [9, 10], hospital teams and units vary in the degree to which interactions between team members produce unpredictable behavior and create new behavior. Also, hospital teams and units differ in the degree to which linear thinking predict results. Thus hospital units differ in their degree of complexity which allowed us to learn the relationships between the complexity, autonomy, and performance orientation in the 31 hospital units. Second, we examine the interaction between complexity and autonomy and complexity and performance orientation to observe how these interactions may be different in hospital settings compared to other industries. Hospital leaders have accelerated their investment in innovation using approaches from the product design, R&D, and technology fields to solve health care system challenges [4, 5]. However, scholars caution health care leaders about adopting approaches from other industries without considering the impact of the organizational context and complexity [17]. Specifically, while high autonomy and low performance orientation may be ideal for innovation in R&D settings, it may be detrimental in healthcare settings. This study is the first, to our knowledge, to explicitly examine autonomy and performance orientation on hospital unit innovation.

### Theory

#### Hospital unit innovation and complexity

Various literatures have argued that organizations need to develop innovation within their organizational units [18]. Units may pursue both the development of new products and services, or build on existing knowledge and extend existing products and services [18, 19]. Innovative units should be cross-functional, probe and seek for help, and face uncertainty of new problems. These characteristics of innovative organizational units make hospitals an ideal setting to examine innovation.

Hospital units are also described as complex. Complexity at the unit level can be due to instability in staffing, instability in patient characteristics and needs, and “complexity compression” or assuming additional, unplanned responsibilities while simultaneously conducting their multiple responsibilities in a condensed timeframe [20, 21]. Thus, the level of complexity that each unit experiences is different. Departmentalization, differing shifts, and different professional hierarchies introduce unit complexities that influence innovation [22]. With varying volumes, case mixes, and functional complexity there is need to understand how unit-level complexity may influence the ability of hospital units to implement innovative practices.

Recently, scholars found that *unit complexity* is associated with negative patient outcomes and system outcomes [21]. Yet, we do not fully understand how complexity might influence hospital unit innovation. Because the influence of complexity on innovation is theorized as a multi-level phenomenon [23], there is a need to understand its influence on innovation not only at the organizational level but at the unit level. Table 1 summarizes a sampling of empirical studies on hospital unit innovation. We find that four phases of innovation that tend to be the focus of such studies: idea generation, idea adaptation to the targeted setting, innovation adoption or implementation in the targeted setting, and innovation spread across multiple units within the same organization. From these example studies, we see that complexity is not typically included in hospital unit innovation studies. Broberg and Edwards [24] do mention that because the hospital unit is a “complex sociotechnical system” comprised of both technical and social components, that the culture between professional domains must be considered when generating innovative ideas. Another study suggests that complexity theory would help in determining predictors for innovation [25].

**Table 1:** Sampling of Empirical Studies of Hospital Unit Innovation

Author, Date	Summary	Innovation Focus	Consideration of Complexity	Factors related to innovation
Broberg and Edwards, 2012	Qualitative/Participative design, user-driven innovation process to develop a novel concept of the spatial and organizational design of an outpatient department in a hospital	Generation	The outpatient department was a “complex sociotechnical system,” that influenced the innovation process in terms of culture and politics in the meeting between different professional domains.	Not included
Su et al., 2011	Action research study of an innovation initiative between the supply department and 155 internal units to reduce unit inventory costs	Generation	Not included, though future research suggests complexity theory may help to determine predictors of logistics innovation in hospitals	buyer-supplier relations
Pearson et al., 2008	Multi-site case study of the strategies used to spread four changes designed to facilitate a smooth, safe, and patient-centered shift change across multiple med-surg units in 3 hospitals	Adaptation and Spread	Not Included	Designation of spread organizers or managers, strategic selection of spread units, designation of nurse champions, clear senior leadership support, collaborative session, communication mechanisms, written spread materials, allocation of resources
Jensen and Chandler, 1994	Survey of 391 employees across 8 hospitals identifying how innovation and restrictive conformity influence personal outcomes	Generation and Adoption	Not included	Personal outcomes associated with innovation: Greater role clarity, organizational involvement, and satisfaction, and lower role conflict and willingness to leave the organization

In studies that examine innovation at the organizational level, we find more examples of the consideration of complexity and how it may relate to the unit level. Table 2 summarizes a sampling of empirical studies on hospital organizational or managerial innovation. We find mixed results; while some studies do not find complexity to be a signification predictor of adoption [26, 27] others do find a correlation [28–30]. We also see that complexity is measured in multiple ways: via teaching hospital status, the number of distinct services or specialties, and involvement in professional activities or training.

**Table 2:** Sampling of Empirical Studies of Complexity and Innovation in Healthcare

Author, Date	Findings related to complexity	Innovation Focus	Measurement of Complexity	Other factors related to innovation and complexity
Cockerill et al., 1999	Complexity was not found to be a significant predictor of adoption of a managerial innovation (resource planning tool)	Adoption	Teaching hospital status	Perceived value and accuracy of innovation, ease of use, resource planning, and physician support
Glandon et al., 1995	Complexity was correlated with the adoption of a managerial innovation (cost accounting systems)	Adoption	Teaching hospital status	n/a
Meyer and Goes, 1988	A combined scale of organizational size, complexity, and strategy (eagerness to penetrate new markets) significantly impacted innovation assimilation	Adoption	Availability of 24 distinct medical services; i.e., horizontal differentiation.	Medical specialization and CEOs as influential proponents of innovation
Hage and Dewar, 1973	Complexity was significantly correlated with the adoption of new programs.	Adaptation and Adoption	Two complexity variables: number of different operational specialties and involvement in professional societies	CEOs and leaders as influential proponents of innovation
Hage and Aiken, 1967	Complexity was correlated with the rate of program change, but not a significant predictor when controlling for other organizational variables (age, size)	Adoption	Three complexity variables- number of different professional specialties, amount of professional training, and the extra-organizational professional activity	Staff attitudes toward change was slightly, but negatively correlated with the rate of program change (−0.14)

Based on these mixed results and calls for more complexity theory to understand predictors of innovation, this study uses a complex adaptive systems (CAS) approach to examine the relationship between complexity and hospital unit innovation. Scholars increasingly apply complex adaptive systems theory to describe hospitals and suggest that these interactions can produce valuable, new, and unpredictable capabilities that are not inherent in any of the parts acting alone [12].

The underlying dynamics influencing CAS include Internal Mechanisms (including the roles of agents and self-organizing or emergent rules), Co-Evolution (including non-linear changes) and the Environment (including dyanamism, or extent and rate of environmental change) [13, 23, 31]. Because this study focuses on the unit level, we focus on Internal Mechanisms that may influence the role of complexity on innovation, namely the role of agents’ development of rules for action and goals and behaviors.

#### Agent rules and autonomy

CAS are “composed of independent agents whose behavior is based on physical, psychological, or social rules” [32]. The complex nature of these healthcare CAS often requires flexibility via self-organization and autonomy to handle medical emergencies and uncertain situations [9, 33]. *Autonomy* is defined as the degree to which an individual is given substantial freedom, independence, and discretion in carrying out a task, such as scheduling work and determining procedures to follow [34].

In tech firms, the capacity to explore for new innovations is often fostered by providing high autonomy [15]. Yet, while service sector workers may require some flexibility in decision making for client-facing processes, they still also require managerial control, i.e., a lower level or balanced level of autonomy, to achieve innovation [14]. While high autonomy may be common in CAS hospital units [9], high autonomy may not be ideal for hospital unit innovation given high workload expectations in addition to any innovations [35], a punitive legal environment [36], and the functional and relational dependencies of medical staff tasks. Formally guiding the innovation process through top-down initiated projects has been found to increase the rate of innovativeness in hospital settings [37], calling into question the extent to which autonomy may be helpful or harmful for innovation in hospital settings.

While the influence of autonomy on the relationship between complexity and innovation has not been explicitly included in hospital unit innovation studies, there have been mixed findings and discussions of managerial control. Some note that looser managerial supervision is common as hospital units pursue innovation [28]. Others find that the designation of specific innovation “spread managers,” champions, and leadership support are strategies for achieving healthcare innovation [38]. Thus, we aim to examine the influence of autonomy on the relationship between complexity and innovation. Our first hypothesis is:

Hypothesis 1: Unit complexity is associated with higher innovation performance when autonomy is low rather than high.

#### Agent behaviors and goal orientation

Within CAS, agents’ goals and behaviors highly influence the system; emerging behavioral patterns arise from adaptation and change [32]. When innovation has been observed within complex healthcare settings, scholars also note performance-oriented behaviors, such as increased boundary scanning and search behaviors, and the development of schema for action [28, 33]. As noted in a qualitative observation from Jansen and Chandler on conformity and innovation [22], “I don’t need people bringing me more problems, but I can really use people bringing me more solutions. (p. 65)” Simply being favorably disposed toward change in one’s personal orientation does not necessarily lead to the adoption of innovations in complex healthcare settings [27]; rather an action-oriented, performance-centric behaviors may be critical to push through the complexity to achieve innovation.

Performance orientation entails wanting to do well and demonstrate competence compared with others or with normative standards, often to attain favorable judgments of ability [39]. Performance orientation is often contrasted with learning orientation, or a desire to master tasks and seek out challenges to gain competence. In many industries, learning orientation may be nurtured, allowing employees to explore multiple areas of inquiry. However, performance orientation may be ideal in certain settings. Many public and not-for-profit organizations, including most health care systems, often have higher levels of bureaucratic control that may inhibit innovativeness [14]. Performance orientation is often associated with recognition, the opportunity to promote one’s ideas in the organization, and other social incentives that overcome innovation barriers [39]. New health care processes and procedures may fail if there is a lack of stakeholder awareness, promotion, and buy-in before and during implementation [40, 41].

The influence of performance orientation on the relationship between complexity and innovation has not been explicitly included in hospital unit-level innovation studies. However, CAS theorists posit healthcare staff tend to be “attracted” to developing and implementing innovations if the staff see the connection between the current innovation and their desire to perform well relative to their targeted patient population [12]. Certain rules and intangible rewards, e.g., believing the innovation was “my” idea, can also attract team members towards certain actions [9]. Thus, we aim to examine the influence of performance orientation on the relationship between complexity and innovation. Our second hypothesis is:

Hypothesis 2: Unit complexity is associated with higher innovation performance when performance orientation is high rather than low.

## Methods

### Ethics

The methods for this study were a part of a larger research effort to understand management practices in hospital units; as such, these methods are summarized from [42]. We distributed questionnaires to the staff of a 450-bed, mid-sized community hospital located in Haifa, Israel, serving 150,000 patients per year. The study was submitted to and approved by the hospital (Bnai Zion, Haifa, Israel) Institutional Review Board committee as exempt. An informed consent letter was on the first page of each questionnaire, in which we explained that participation is voluntary and we would not refer to any individual responses. Consent from study participants was verbal. Questionnaires responses were anonymous.

### Participants

We distributed the questionnaire to staff members across 31 units. We targeted and aquired a response rate of 20% for 16 units with less than 20 staff members, and 10% for 15 units with more than 20 staff members [43]. The study comprised 17 medical units: anesthesiology, cardiology, emergency department, gastroenterology, general intensive care, internal medicine unit (two wards, A and B), internal medicine unit C/urology, obstetrics and gynecology, occupational therapy, ophthalmology, orthopedics, pediatrics, pediatric surgery, recovery room, rehabilitation, and surgery. We also included fourteen service: blood bank, chemistry, endocrinology, genetics, immunology, infectious diseases, microbiology, nephrology, pathology, pharmacy, radiology, radiology (radio-isotope scanning), reception, and social services. 163 front-line staff completed the independent-variable questionnaire, with an average of five staff members from each unit. Sixty-nine upper-level staff (different staff from those who completed the independent-variable questionnaire), two to three from each unit, completed the performance questionnaire. We attained a 95% the response rate for the dependent variable.

### Measures

We used a 5-point Likert scale, ranging from “Not at all” to “A great deal” for all of the survey items (Appendix A). We used previously tested instruments for unit complexity, autonomy, performance orientation, and innovation performance; all except innovation performance had been previously tested in a healthcare setting. Unit complexity was tested in hospitals by [44], autonomy was tested by [45], and performance orientation was tested by [46].

Four items for unit complexity were adapted from [47]. An example item is “Your group’s environment is changing.” Autonomy was a moderator and evaluated through four items adapted from [45]. An example item for autonomy is “Our department can decide when to start each of our tasks.” Performance orientation was a moderator and evaluated through four items adapted from [48]. An example item for performance orientation is “Our department would like to show we can perform well.” These were measured through the independent-variable questionnaire.

The scale of the variables of unit complexity, autonomy, and performance orientation exhibited a sufficiently strong agreement (median rwg = .82, .90, .92, accordingly), and significant between-group variance (tested by one-way ANOVA, F (25, 115) = 4.94, *p* > .05, F (25, 118) = 1.84, *p* > .05, F (25, 117) = 1.79, *p* > .05, accordingly). Intraclass correlations were ICC(1) = .42, .09, .08 and ICC(2) = .80, .35, .33 accordingly. The interclass correlation values in our study were consistent with those in previous team research and considered acceptable for justifying the aggregation [49]. Specifically, for ICC(1), values of .05 and higher, which are based on a significant one-way ANOVA test, are considered acceptable for justifying the aggregation [49, 50].

Innovation performance relates to the generation of new ideas and their implementation [3, 51]. An example item for innovation performance is “The department implements innovative ideas”. This was measured through the dependent-variable questionnaire. Having team managers evaluate the unit’s innovation performance is common in the innovation literature e.g., [3]. We asked at least two respondents per unit for the dependent variable increases the agreement and reliability of the dependent variable sample. Also, by sampling the upper-level staff, they are experts and are very familiar with the department.

Since the study comprised medical units and service units, we controlled for unit type (“medical” = 0, “service” = 1). This was measured through the independent-variable and dependent-variable questionnaires. We controlled for the unit type because we wanted to reduce the potentially confounding relationship between unit type and our independent variables. For example, medical units may have more autonomy than service units, but we did not want this potentially confounding relationship to influence the regression.

### Data collection procedures

Before conducting the survey, we had several meetings with the hospital leadership and staff members to gain buy-in and build trust for the data collection which was done by hand with paper surveys. We also gained an understanding of some of the kinds of hospital unit innovations through these initial interviews, e.g., procedural and equipment innovations.

We collected data for the independent-variable questionnaires from general hospital staff and collected data for the dependent-variable questionnaire from two to three senior staff members, i.e., unit heads or senior ranking staff. By having different data sources for the independent and dependent variables, multiple respondents per team, and previously tested instruments, we decreased our potential for bias [52]. Research assistants collected all data during the main working hours (9a-5 pm). Participants could either complete the questionnaire completely, or the research assistants would leave the questionnaire and collect it later.

We chose to study the research question conducting a survey based on guidelines published by Edmonson and McManus which discusses how to assess the appropriate fit between existing knowledge and the chosen methodology for a new study [53]. Because there was existing qualitative knowledge from previous studies of healthcare innovation that helped to build theory in the area, the use of a questionnaire is a suggested approach for theory testing. Moreover, since we wanted to compare innovation antecedents in health care based on acceptable antecedents in other industries, the use of questionnaire allows us to compare our results with earlier approaches in other industries.

## Results

### Level of analysis

The hospital unit level was the unit of analysis for all variables. We aggregated individual responses to the unit level to create a unit mean for each variable. We calculated the mean score of the innovation performance for each unit by averaging the corresponding two (or three) managers’ means scores. Table 3 summarizes the means, standard deviations, and correlations among the variables. The correlations were between 0.3 and 0.7.

**Table 3:** Means, Standard Deviation, and Correlation ^a,b^

	Mean	SD	1	2	3	
1. Unit complexity	3.26	0.71				
2. Performance Orientation	4.42	0.30	0.46**			
3. Autonomy	4.05	0.38	0.32*	0.51**		
4. Innovation	3.91	0.49	−0.11	0.19	−0.18	

^a^These statistics are at the unit level of analysis

^b^Cronbach’s alpha (α) coefficients appear in square brackets

*n* = 31

* *p* < 0.05 ** *p* < 0.01

### Confirmatory factor analysis

The confirmatory factor analysis (CFA), in constructing the unit characteristics factor structure of autonomy, performance orientation, and unit complexity, yielded acceptable fit levels (χ2(30, 157) = 60.28, *p* = 0.0009; GFI = 0.92; RMSEA = 0.08; NFI = 0.91; NNFI = 0.94; CFI = 0.95). A one-factor model was created to validate these results (χ2(27, 157) = 362.97, *p* < 0.0001; GFI = 0.67; RMSEA = 0.28; NFI = 0.42; NNFI = 0.26; CFI = 0.44). The chi-square difference between the one-factor model and the three-factor model, χ2(2, 144) = 302.69, *p* < 0.0001, significantly indicate the poorness of fit for the one-factor model relative to the three-factor model.

### Hypotheses testing

To test the hypotheses, we regressed innovation performance on the control variable, the three independent variables, and the two two-way interactions hypothesized earlier. The model (see Table 4) presents the innovation performance results. The two-way interaction between unit complexity and autonomy was significant. In addition, the two-way interaction between unit complexity and performance orientation was also significant. To test the interactions, we used standardized the data between − 1 and 1. To understand the nature of the significant interactions in this model we followed the graphing method outlined by [54].

**Table 4:** Results of Linear Regression

Innovation Performance			
	Model 1	Model 2	Model 3
**Intercept**	.06 (.25)	.31 (.26)	.40 (.57)
Unit Type	−.14 (.37)	−.69 (.44)	−.44 (.37)
Unit complexity		−.46 (.25)^*^	−.42 (.21) ^✝^
Autonomy		−.34 (.20)	−.77 (.20)^***^
Performance Orientation		.59 (.22)^*^	.84 (.20)^***^
Unit complexity * Autonomy			−.42 (.15)^*^
Unit complexity * Performance Orientation			.82 (.21)^***^
Model Statistics			
R^2^	.00	.25	.53
F	0.15	2.15	4.60**

*n* = 31

^✝^*p* < 0.1

* p < 0.05

** *p* < 0.01

*** *p* < 0.001

**Fig. 1:**
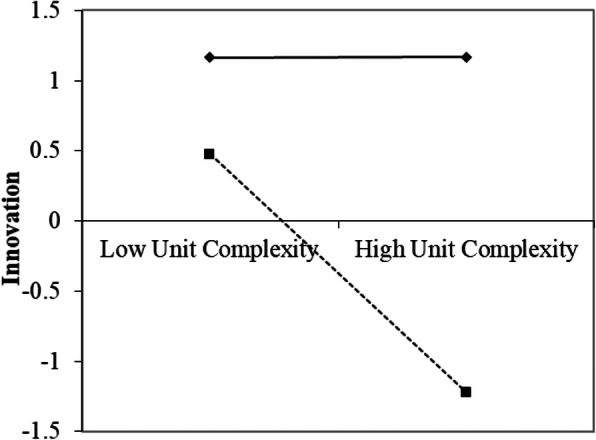
Linear Regression Lines of Innovation as a Function of Unit Complexity and Autonomy

The results of the simple slope of the interaction between unit complexity and autonomy (Fig. 1) show that when autonomy was high there was significant support for the assertion that the higher the unit complexity, the lower the innovation performance (b = −.62, t(25) = − 3.34, *p* < .01). However, when autonomy was low, higher unit complexity was not associated with a reduction in the innovation performance (b = 0.02, t(25) = .09, *p* > .1). Therefore, Hypothesis 1 was supported.

**Fig. 2:**
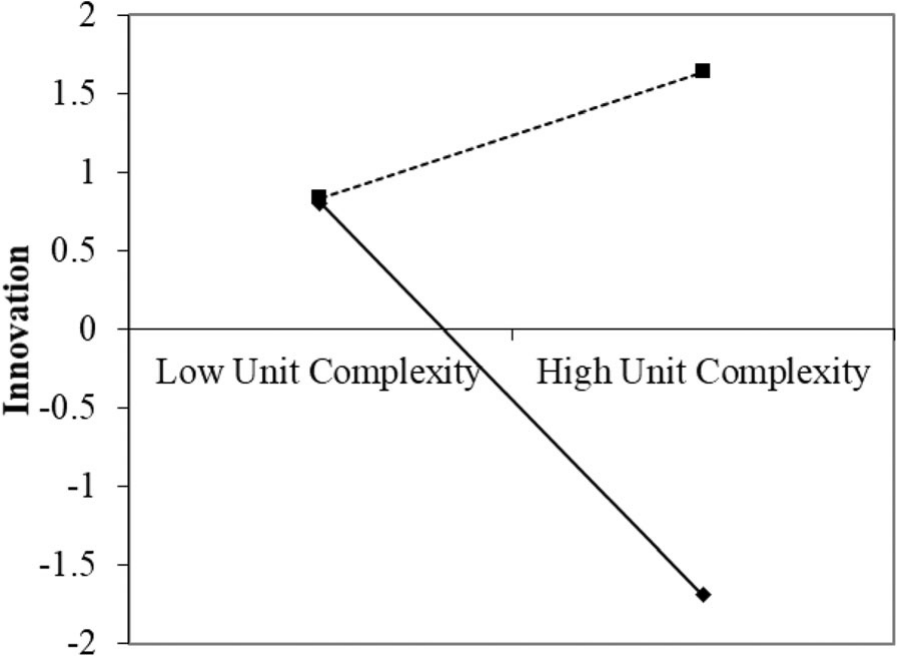
Linear Regression lines of Innovation as a function of Unit complexity and Performance Orientation

The results of the simple slope of the interaction between unit complexity and performance orientation (Fig. 2) show that when performance orientation was low there was significant support for the assertion that the higher the unit complexity, the lower the innovation performance (b = −.92, t(25) = − 3.7 *p* < .01). However, when performance orientation was high, higher unit complexity was not associated with a reduction in the innovation performance (b = 0.32, t(25) = 1.45, *p* > .1). Therefore, Hypothesis 2 was supported.

## Discussion

Two main contributions are at the core of this study. First, unit complexity is associated with higher innovation performance when autonomy is low rather than high. This is contrary to the prevailing view of innovation from other industries that focuses on high autonomy to promote innovation, e.g., [55].

This result suggests that, given the high unit complexity of many hospital units, less autonomy is best to achieve innovation performance. The lowest innovation performance was found when both autonomy and unit complexity were high. This seems intuitive for health care though not in other sectors: with high complexity, having a department with extreme freedom and independence to dictate their tasks may lead to the “chaos” trap where it is unclear who is responsible for key innovation goals [56]. When autonomy is high and unit complexity is low, innovation is also significantly lower than when autonomy is low and unit complexity is low. This suggests that having some dependence or guidance on rules and procedures is still beneficial in a relatively simple or predictable environment. Studies show that having standards and rules is helpful [57], particularly when complexity is low [58]. If the work environment is predictable and one can use the best existing knowledge, then low autonomy is a good way to instruct individuals rather than letting them reinvent the wheel. It may also be that for hospitals, if unit complexity is low, then there is no drive for innovation over the status quo.

The best scenario is when unit complexity is high and autonomy is low. Our findings align with previous research that find the designation of specific innovation “spread managers,” champions, and leadership support are strategies for achieving healthcare innovation [38]. For example, a hospital may choose to have an innovation division or group that works alongside hospital units to support their innovation efforts [4]. Having low autonomy simply infers that these unit providers are gaining guidance from leadership as to what innovations to pursue and implement. This finding is at the heart of finding innovative practices that specifically apply to health care: where autonomy is typically a detriment to innovation, in this case, having less autonomy to choose tasks may lead more innovation.

Second, unit complexity is associated with higher innovation performance when performance orientation is high rather than low. This result suggests that given the high unit complexity of many hospital units, a stronger performance orientation among department members is best to achieve innovative performance. The lowest innovation performance was found when unit complexity was high and performance orientation was low. This seems intuitive for health care though not in other sectors: if the unit’s work is complex, having a department with no desire to show that they can perform well or no desire to seek to achieve success may not have the drive or incentives necessary to wade through such high unit complexity and achieve innovative performance [14]. When performance orientation is low and unit complexity is low, innovation is also significantly lower than when performance orientation is high and unit complexity is low. This suggests that having some desire to work on assignments that can demonstrate a department’s capabilities and seeking to achieve success is still beneficial in a relatively predictable environment.

The best scenario is when unit complexity is high and performance orientation is high. These relationships echo Plesk and Wilson’s [12] propositions on the relationship between complexity and performance orientation: health care staff are above all interested in providing treatment to their patients. Their innovation has a direct influence on their current work and proving the success of an idea, as opposed to an approach to innovation that may value the potential gains of some good ideas prior to implementation or proof of success. This finding also further emphasizes the findings of Alexander and Van Kippenberg’s propositions [39] that the promotion of ideas is necessary for innovation in health care.

We showed that unit complexity is associated with higher innovation performance when autonomy is low rather than high and when performance orientation is high rather then low. This is a counterintuitive view of innovation because it is the opposite of what is viewed as “best practices” in other industries. Studies in other industries, e.g., R&D and technology, typically find that high autonomy and low performance orientation promote innovation [3, 58]. Because health care tends to adopt innovation practices from these industries, we caution healthcare leaders to avoid mimicking all behaviors and approaches from these industries, but rather to follow an approach that is best for hospital units given that they are CAS.

### Implications for practice

Our study encourages hospital leaders, hospital unit managers, and health care policy makers to view hospital unit innovation through a CAS lens. Recognizing the CAS Internal Mechanisms within hospital units, namely the role of agents’ development of rules (as understood through autonomy) and agents’ goals and subsequent behaviors (as understood through performance orientation) helps us to design more successful innovation programs. Also, while hospital unit managers may borrow ideas from other service and production sectors about how to promote innovation, our results show that following these practices for autonomy and performance orientation may actually harm the innovation in the hospitals. Our counterintuitive results thus are important for achieving innovation in hospitals. Taken together, these two contributions lead us to the following practical guidelines for hospital unit managers to achieve innovation for top-down innovation initiatives, bottom-up innovation initiatives, and innovation training programs.

First, for top-down innovation initiatives initiated by hospital directors or C-suite leadership, we suggest that leaders consider incentivizing innovation programs in order to encourage the achievement of the innovation goals. We see that many healthcare innovation initiatives are incentivized via governmental mechanisms, e.g., the Center for Medicare and Medicaid Services Innovation Program in the United States [59], and academic organizations, e.g., MIT Hacking Medicine challenge [60]. We encourage similar approaches to be used within hospital units on a smaller scale. Incentives should include recognition so that team members can share their performance and innovation to others. Second, for bottom-up innovation initiatives initiated through observation from an individual team member or small group of team members within a unit, we suggest that unit leaders not only encourage such innovation, but provide some boundaries around how the innovation is prioritized, the time span for innovation, how the resultant changes are implemented, and how to best share the findings with other relevant units. Based on our findings, this will give the team members the additional support needed to navigate the unit complexity while still executing on the innovation idea. Finally, as healthcare systems develop innovation training programs, we encourage them to not simply teach design thinking or new product and service techniques “out of the box” from R&D and tech. Rather, these techniques should be taught within the context of the hospital units. So for example, for design thinking training, as a group ideates or develops and tests prototypes of the new product or service, they may wish to have several rounds of input from leadership so that the right level of prioritization and change implementation support can occur.

### Limitations and future research

The present study benefited from a high unit-level response rate and a research design that allowed the linkage of the independent variables’ data and the performance data that was assessed separately. Yet the study has four limitations we would like to point out. First, though acceptable in similar research designs [61], it has a relatively small within-unit sample size. Second, the study is a cross-sectional research that was conducted over a short period. Third, this study uses a broad definition of innovation that includes procedural, process, or product innovations. We did not have access to objective or administrative data by which to assess innovation performance or to add additional personal or organizational characteristics as control variables. Fourth, we did not develop a measure of complexity specifically for hospital units.

These limitations suggest interesting future research directions. Future research can consider a longitudinal research design and, where possible, more objective measures of innovation performance where available, e.g., patents, technology transfer records, or number of new service lines. Future research can also make the distinction between innovation types within hospitals and potentially work with a growing number of innovation departments within hospitals to properly codify and measure innovation types. Future research can also develop a new psychometric for complexity that is specific to hospitals. In order to determine to what extent the 31 hospital units meet the criteria of adaptive systems we used the well established Lawrence and Lorsch’s complexity measure from their study of differentiation and integration in complex organizations published in Adminsitrative Science Quaterly (ASQ) [47]. The more recent research of Pype et al. [9] could lead to the development of a psychometric specifically for hospital unit complexity, considering all of the potential items of CAS hospital units. In order to use it, future resaerch should test the accuracy and validity of the metric. It is likely that some items within the CAS team assessment will need to be separated to be valid and reliable as a psychometric, e.g., autonomy and attractors. Our study can help in this direction in comparing the results of the new measure to that of the existing one from Lawrence and Lorsch [47].

Finally, future research could examine specific approaches to innovation that are from other industries, e.g., the use of “skunkworks”-like teams, design thinking initiatives, and hack-a-thons. These studies should consider a mixed method approach, collecting common measures via surveys and also elaborating on our results and take additional steps by collecting qualitative data as well.

## Conclusion

In conclusion, this study moves beyond the common edict that, “healthcare is complex” to examine *how* complexity influences innovation performance and what healthcare providers and administrators can do to innovate within complex units. Our study suggests a message that may be unintuitive for other industries but highly intuitive in health care. In health care, in order to achieve high levels of innovation, departments with higher levels of unit complexity should respond with lower staff autonomy and higher emphasis on performance orientation. In other words, the more complicated it is to provide treatment to patients (i.e. complexity is high), then less freedom and independence should be given to hospital staff (i.e. low autonomy), and more emphasis on proving the department’s capabilities to treat patients (performance orientation) lead to more innovation. Healthcare leaders should implement minimum specifications to limit autonomy and drive innovation. Healthcare leaders should also find ways to link innovation to overall patient-centered performance to “attract” healthcare providers to innovation. In other words, innovation for innovation’s sake is more of a stick than a carrot in healthcare. Innovation becomes attractive when it is aligned with a unit’s desire to be successful and is tied into their overall objectives and desire to succeed.

In health care, innovation is a result of emphasizing the actual problems, here and now, while in other industries being a “dreamer” may increase innovation. The complexity of health care is such that simply “dreaming” or “ideating” will not achieve innovation [62]. However, when health care staff members emphasize the promotion and implementation of actual assignments tied to successfully treating patients, they achieve high innovation. Hospitals looking to implement innovation programs should provide guidance to teams and departments regarding the type of innovation sought and provide support in terms of time and management commitment. Hospitals should also find ways to promote and make successful implementations of such innovations visible in the organization.

### Acklowledgements

We acklowledge Qing Li for her support on the data collection and John Carroll for his support in the early research conceptualization. Part of the material in this manuscript was presented at the Production and Operations Management Society Conference (POMS) annual conference held on May 2–6 in Washington, D.C., USA .

## Data Availability

Data is available upon request to the corresponding author.

## Appendix A

### Innovation*:* Adapted from [3].

To what extent does this department:

1. Suggests innovative ideas.
2. Initiates innovative work processes.
3. Implements innovative ideas.
4. Use the most innovative methods/processes.
5. Adapts new and innovative technologies.

### Autonomy [45].

Our Department:

1. Can prioritize our tasks
2. Can decide when to start each of our tasks
3. Has the freedom to implement changes
4. Is independent in doing our work

### Hospital Unit Complexity: Adapted from [47].

Our department:

1. Is unpredictable.
2. Is changing.
3. Is complex.

### Performance orientation: Adapted from [48].

Our department:

1. Would like to show we can perform well
2. Prefers to work on assignments where we can prove our abilities to others
3. Seeks to achieve success in our work
4. Aims to get the job done

## Abbreviations

CAS: Complex adaptive systems
ANOVA: Analysis of variance
ICC: Intraclass correlations
GFI: Goodness of fit index
RMSEA: Root mean square error of approximation
NFI: Normed fit index
NNFI: Non-normed fit index
CFI: Comparative fit index
